# Retracted and republished from: “The current state of research on influenza antiviral drug development: drugs in clinical trial and licensed drugs”

**DOI:** 10.1128/mbio.00175-24

**Published:** 2024-03-29

**Authors:** Yanbai Li, Shanshan Huo, Zhe Yin, Zuguang Tian, Fang Huang, Peng Liu, Yue Liu, Fei Yu

**Affiliations:** 1 Hebei Key Laboratory of Analysis and Control of Zoonotic Pathogenic Microorganism, Hebei Wild Animal Health Center, College of Life Sciences, Hebei Agricultural University, Baoding, China; 2 Department of High-Tech Development, Baoding City Science and Technology Bureau, Baoding, China; 3 Epidemic Prevention Laboratory, Tongzhou District Center For Animal Disease Control and Prevention, Beijing, China; 4 Department of Biochemistry and Biophysics, University of California, San Francisco, California, USA; Albert Einstein College of Medicine, Bronx, New York, USA

**Keywords:** influenza virus, antiviral drugs, mechanism of action, clinical drugs, adverse event

## Abstract

Influenza viruses (IVs) threaten global human health due to the high morbidity, infection, and mortality rates. Currently, the influenza drugs recommended by the Food and Drug Administration are oseltamivir, zanamivir, peramivir, and baloxavir marboxil. These recommended antivirals are currently effective for major subtypes of IVs as the compounds target conserved domains in neuraminidase or polymerase acidic (PA) protein. However, this trend may gradually change due to the selection of antiviral drugs and the natural evolution of IVs. Therefore, there is an urgent need to develop drugs related to the treatment of influenza to deal with the next pandemic. Here, we summarized the cutting-edge research in mechanism of action, inhibitory activity, and clinical efficacy of drugs that have been approved and drugs that are still in clinical trials for influenza treatment. We hope this review will provide up-to-date and comprehensive information on influenza antivirals and generate hypotheses for screens and development of new broad-spectrum influenza drugs in the near future.

## INTRODUCTION

Influenza is an acute, contagious respiratory illness, caused by influenza viruses (IVs), that presents an increasing public health burden (1, 2). IV is a negative-sense, single-stranded RNA (ssRNA) virus of the Orthomyxovirus genus, characterized with high genetic variability, and has circulated in the human population at least since 1580 (3). IV is classified as A, B, C, and D according to the antigenicity of nucleoproteins (NPs) and matrix (M) proteins *in vivo* (4). The genome of IV consists of eight ssRNA segments for influenza A and B viruses (IAV and IBV) and seven ssRNA segments for influenza C virus (ICV). These ssRNA segments are each complexed with the viral RNA-dependent RNA polymerase (RdRp) and NP, forming the viral ribonucleoprotein (vRNP) (1), which is packaged into viral particles. IAV and IBV infect humans with high morbidity and mortality, causing severe economic losses (4). Children are the main infection population of ICV, which can cause mild respiratory diseases. There are no reports of influenza D virus (IDV) infection in humans. Seasonal influenza is the main cause of worldwide pandemic flu.

According to the World Health Organization data, about 3–5 million cases of serious illness and 290,000–650,000 deaths from respiratory diseases are related to seasonal influenza in the world every year (4). In addition, highly pathogenic avian influenza, once they acquire the ability to be transmitted from human to human, can also pose a serious threat to human health. It is mainly spread between poultry and wild birds. People could become infected avian IVs when they contact with carrying or diseased poultry (5). Most recently, the main avian IVs that can infect humans include the H5, H7, and H9 subtypes (5). The U.S. Center for Disease Control and Prevention (CDC) recommends oseltamivir, a neuraminidase inhibitor, as the primary treatment drug for avian influenza (6).

## CURRENT STATUS OF IV CONTROL

Influenza vaccines have dramatically decreased the number of influenza cases each year (4). However, due to the weak subjective prevention and control awareness, particularly in high-risk populations, and potential inaccurate prediction of the circulating strain, the influenza vaccine alone is not sufficient (7). Influenza infections are further complicated by joint bacterial pneumonia and/or cytokine storm which may further complicate drug treatment. In addition, IV may be overlooked if it occurs simultaneously with other respiratory viruses. Therefore, in the absence of timely prevention and lack of immunity, especially when influenza patients with severe illnesses are infected with seasonal IVs, new influenza strains or pandemics occur; therefore, the treatment and emergency prevention of influenza antiviral drugs are particularly important (7, 8).

From the development of the first anti-influenza drug amantadine to the more recent RNA polymerase inhibitor baloxavir marboxil, currently, only two classes of drugs are Food and Drug Administration (FDA) approved and recommended for use (9). Meanwhile, there are several influenza antiviral drugs in development, some of which have completed clinical trials, with different mechanisms of action and different inhibitory effects (8). These drugs are required to be highly broad-spectrum and safe in order to have a chance of being licensed. As new drugs are approved, the threat of influenza to special high-risk groups will continue to decrease. In this review, we will describe the anti-influenza viral drugs that have been marketed and located in the clinic with reference to the different stages of the IV life cycle, aiming to provide references and suggestions for subsequent drugs development and the emergence of new therapies.

## DRUG CANDIDATE TARGETS BASED ON VIRAL STRUCTURE AND LIFE CYCLE

**Fig 1:**
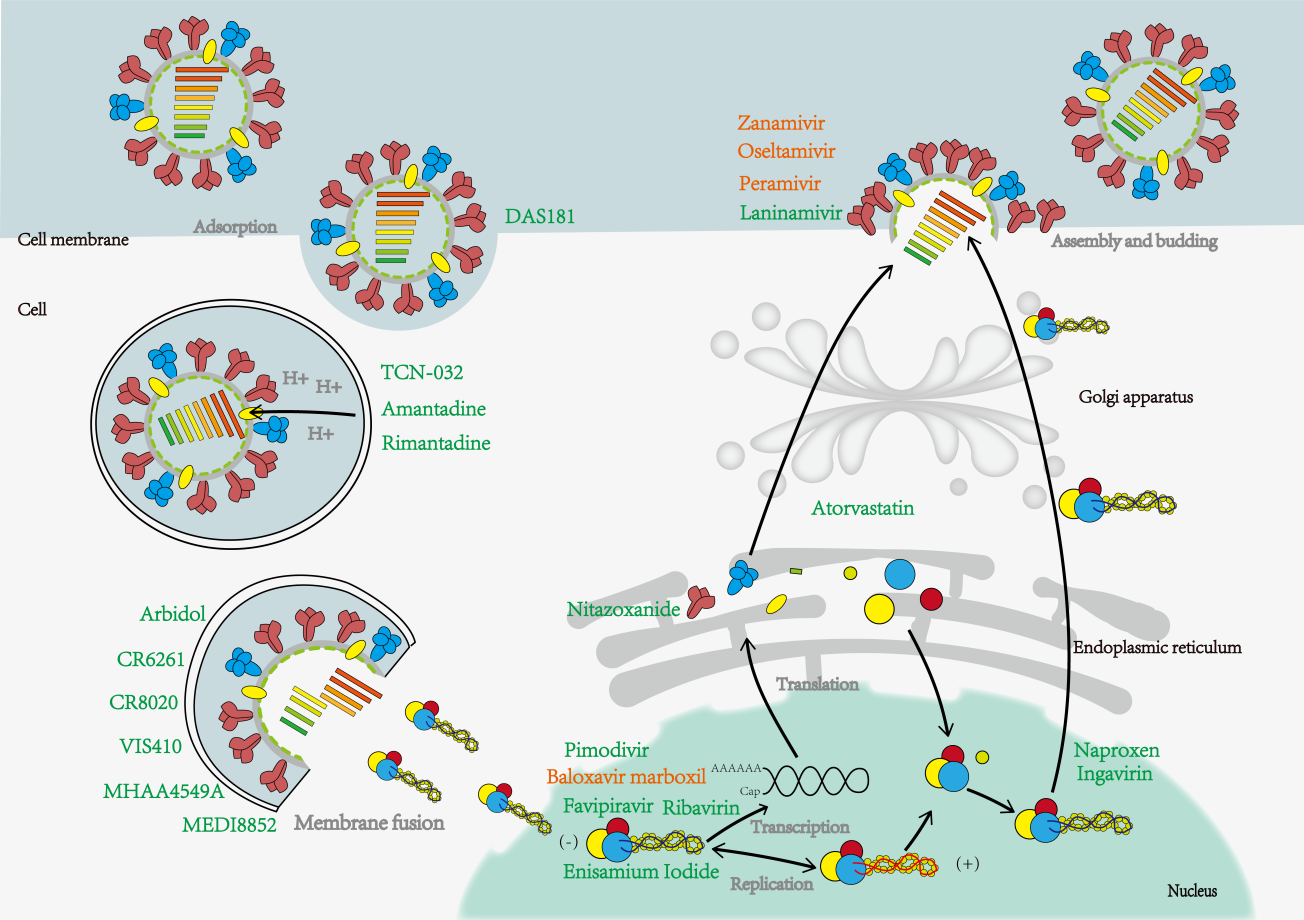
IV infections life cycle and associated drug action stages contain currently approved and unapproved drugs by the FDA, with approved drugs shown in orange font and others in green. IV contacts sialic acid on host cells through surface HA and encapsulates virus particles in endosomes by endocytosis. In the low pH environment, H^+^ enters the virus through the M2 ion channel, and under the action of the fusion peptide, the viral membrane fuses with the endosome membrane, releasing the viral genome into the cytoplasm and then into the nucleus. The viral RNA is transcribed, copied, and translated by RNA polymerase. Finally, it is assembled on the surface of the cell membrane and hydrolyzes sialic acid with neuraminidase (NA) to help release the mature progeny virus.

Although the genome of IV is simple, all viral proteins have non-redundant roles in the infection process and complete the entire replication cycle together. Therefore, these specific proteins can be used as targets for drug design or screening (Fig. 1; Tables 1 to 3).

**TABLE 1:** FDA-approved influenza drugs (include no longer recommended and currently recommended)

Name	Target	Usage	Applicable people (treatment/prophylaxis)	Against	Recommended dosage (adults)	Reference
No longer recommended						
Amantadine	M2 ion channel	ral	−^*a*^	IAV	−	(10–19)
Rimantadine	M2 ion channel	ral	−	IAV	−	
Recommended						
Zanamivir	NA active site	Inhaled	People 7 years and older/people 5 years and older	IAV and IBV	Treatment: 10 mg twice daily for 5 days; Prophylaxis: 10 mg once daily for 7 days	(20–26)
Oseltamivir	NA active site	ral	People 2 weeks and older/people 1 year and older	IAV and IBV	Treatment: 75 mg twice daily for 5 days; Prophylaxis: 75 mg once daily for 7 days	(10, 27–35)
Peramivir	NA active site	Intravenous	People 2 years and older/-	IAV and IBV	A single dose of 600 mg, >15 min	(10, 20, 36–41)
Baloxavir marboxil	PA	ral	People 5 years and older/-	IAV and IBV	<80 kg: a single dose of 40 mg; >80 kg: a single dose of 80 mg	(42–50)

^*a*^ "−" indicates that no relevant information is available at the time of publication.

**TABLE 2:** Other countries’ approved influenza drugs

Name	Target	Usage	Country/time	Applicable people (treatment/prophylaxis)	Against	Recommended dosage (adults)	Reference
Arbidol	HA Stalk	ral	Russia, 1993; China, 2006	People 5 years and older/people 5 years and older	IAV and IBV	Treatment: 200 mg four times daily for 5 days; Prophylaxis: 200 mg twice a week for 3 weeks	(51–64)
Favipiravir	PB1	ral	Japan 2014	Adults	IAV, IBV, and ICV	Day 1: 1,600 mg twice daily; Days 2–5: 600 mg twice daily	(65–73)
Laninamivir	NA active site	Inhaled	Japan, 2010	People 5 years and older/-	IAV and IBV	A single dose of 160 mg	(74–83)
Ingavirin	NP	ral	Russia, 2009	People 13–17 years	IAV and IBV	Treatment: 60 mg once daily for 5 days	(84–87)

**TABLE 3:** Influenza drugs in clinical trials (peptides and small molecule drugs)

Name	Mechanism of action	IC_50_ (μg/mL)	100% Protection *in vivo*	Adverse event	Usage	Clinical studies^*a*^	Reference
Target the host							
DAS181	Mimic sialic acid	0.04–0.9 nM	Pre: 0.3 U/treat/day Post: 30 U/treat/day	Elevated alkaline phosphatase	Inhalation	I NCT00527865, October 2007 to January 2009; I NCT01651494, August 2011 to September 2012; II NCT01037205, December 2009 to September 2011	(88–90)
Atorvastatin	Inhibition of lipid droplet formation in the viral envelope	−^*b*^	−	−		II NCT02056340, October 2013 to June 2018	(91, 92)
Nitazoxanide	Affects maturation of HA	0.31–1 μM	120 mg/kg/day	Headache	ral	II and III NCT01227421, December 2010 to May 2011; II NCT02057757, February 2014 to January 2018	(93–102)
Target the virus							
Ribavirin	Inhibition of RdRp activity	5.1	−	Nausea, vomiting, and diarrhea	ral	I and II NCT00867139, March 2009 to January 2010; II NCT01227967, September 2010 to March 2017	(8, 103–108)
Pimodivir	Inhibition of RdRp activity	0.13–3.2 nM	20 mg/kg twice daily	Diarrhea	ral	IIa NCT01561807, March 2012- ; IIb NCT02342249, December 2014 to May 2016; II NCT02532283, December 2015 to March 2017; III NCT03376321, January 2018 to April 2020	(109–113)
Enisamium iodide	Inhibition of RdRp activity	−	−	−	ral	II and III NCT04682444, April 2009 to May 2010	(114, 115)
Naproxen	Inhibiting NP binding to RNA	16.7–19.2 μM	10 mg/kg/day	No	ral	IIb and III NCT04315194, December 2017 to December 2019	(116, 117)
XC221	−	−	−	−		I NCT03459391, May 2017 to September 2017; II NCT03455491, February 2018 to June 2018; III NCT05544916, August 2022 to May 2023	−
JNJ4796	Inhibition of membrane fusion	0.012–3.24 μM	10 mg/kg twice daily		ral	−	(118)

^*a*^ NCT, clinical trial number.

^*b*^ "−" indicates that no relevant information is available at the time of publication.

Hemagglutinin (HA) is a glycoprotein on the surface of IV, consisting of three identical HA monomers to form a trimer structure (119). According to genetic and antigenic variation, the IAV HA is divided into group 1 (H1, H2, H5, H6, H8, H9, H11, H12, H13, H16, H17, and H18) and group 2 (H3, H4, H7, H10, H14, and H15) (120). HA can be divided into head and stalk parts. In the case of human influenza, the receptor-binding sites located in the head will preferentially target the α2,6-linked sialic acid attached to the galactose of the glycan receptor located on the human respiratory epithelial cell surface, which then encapsulates the virus particle in endosomes through clathrin-mediated or other means of endocytosis, such as micropinocytosis (121, 122). Next, the M2 ion channel opens and guides H^+^ into the virus core, causing weak acidification of the virus core (123). Under low-pH conditions, fusion peptides located in the HA stalk can mediate the fusion of viral and host membranes (121, 124). In the meantime, the interaction between M1 and vRNP is disrupted, allowing the RNP to be released and transported to the nucleus (123, 125).

Next, through the action of RdRp, Virus RNA (vRNA) undergoes transcription, replication, and translation. RdRp is a protein complex consisting of three subunits, including acidic protein (PA), basic protein 1 (PB1), and basic protein 2 (PB2) (126). The transcription of IV requires primers; the PB2 subunit can recognize the Cap structure (7-methyl GTP) of the host mRNA and then use the endonuclease activity of the PA subunit to cut about 8–14 bases downstream of the Cap structure. The base sequence is used as a primer, and the viral mRNA is synthesized using PB1 with RNA polymerase function (1, 126). In contrast to the transcription process, IV replication does not require primers, and RNA polymerase uses vRNA as a template to synthesize cRNA, followed by the synthesis of vRNA using cRNA (1, 126).

Subsequently, various structural and non-structural viral proteins are synthesized on the ribosomes, and proteins involved in RNP assembly are returned to the nucleus for assembly and transported to the surface of the cell membrane along with other protein structures (116). Finally, in the presence of neuraminidase (NA), the virus leaves the host in a budding manner (74). NA is a tetrameric structure, and in IAV, NA is classified into 11 subtypes, which is an important basis for the classification of IAV subtypes, along with HA (120). It is noteworthy that only the N1–N9 isoforms are active in hydrolyzing sialic acid (127, 128). Compared with HA, NA has less evolutionary pressure and is a good target for drug action. Small molecules and antibody drugs targeting NA have shown excellent broad spectrum and protective effects against different subtypes of IV (27, 129, 130).

## APPROVED DRUGS FOR INFLUENZA

At present, several anti-influenza viral drugs have been approved by the FDA or other countries and can be divided into three categories according to their mechanisms of action, including inhibiting viral entry into host cells (HA inhibitor and M2 inhibitors), inhibiting viral replication (RdRp inhibitors), and inhibiting viral particle release from infected cells (NA inhibitors; Fig. 1 Tables 1 and 2) (131). The availability of these drugs has greatly reduced the duration of illness, alleviated the disease, and made an outstanding contribution to suppressing the spread and outbreak of influenza.

### Entry inhibitors

The IV entry inhibitors mainly contain HA inhibitors and M2 inhibitors, of which the M2 inhibitor amantadine was also the first anti-influenza drug approved by the FDA. However, the resistance of IVs to these inhibitors increased dramatically during their use, leading to drug failure, and they are no longer recommended for subsequent use. However, they can still be investigated as drug candidates against emerging IVs.

#### HA inhibitors

**Fig 2:**
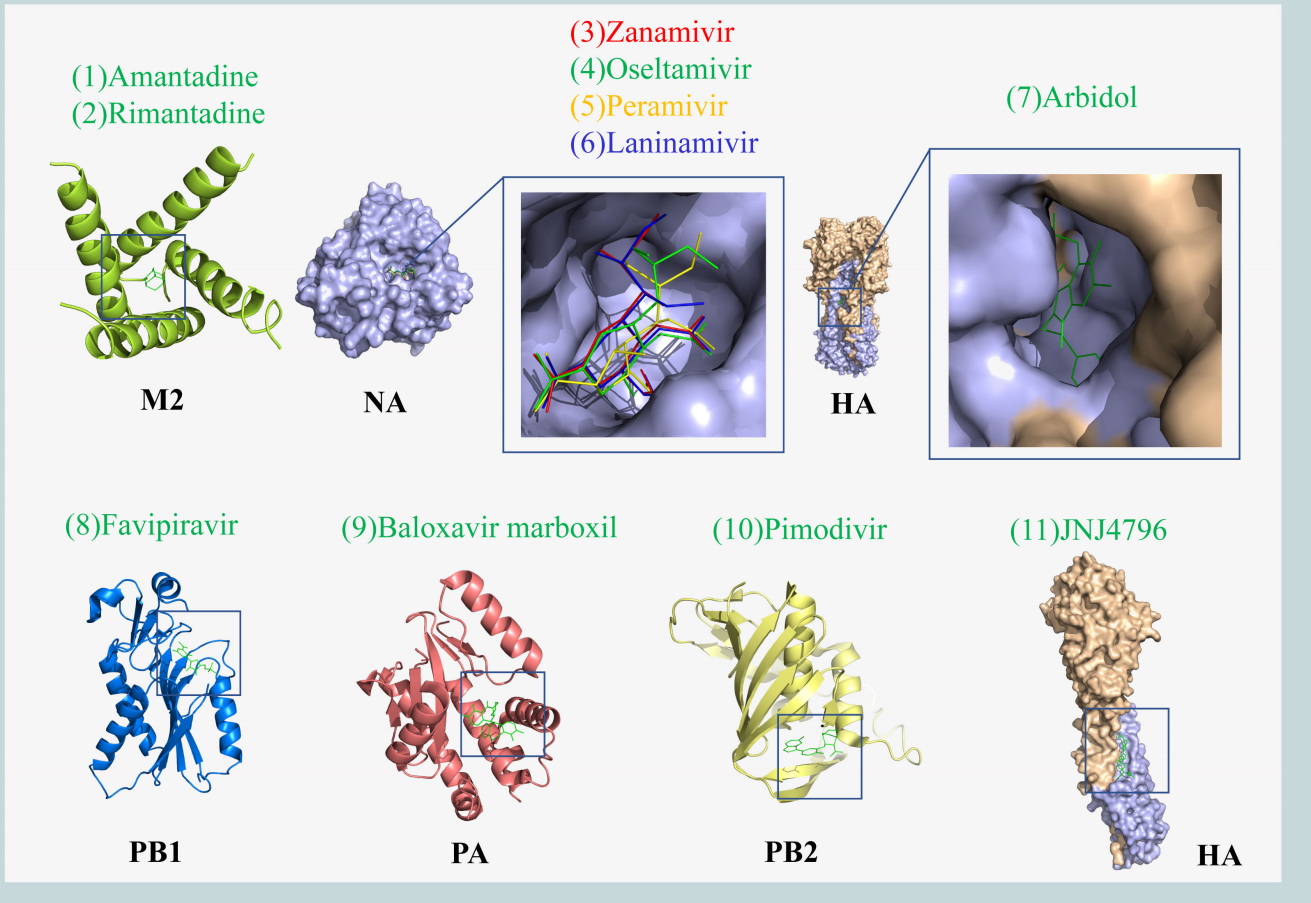
Crystal structures of small molecule drugs of IV. The binding sites of small molecules and related subunits are shown in the box. M2 protein and Amantadine complex (PDB code: 3C9J), NA and Zanamivir, Oseltamivir, Peramivir, Laninamivir complex (PDB code: 4MWR, 4MWW, 4MX0 and 4MWY), HA and Arbidol, JNJ4796 complex (PDB code: 5T6S and 6CFG), PB1 and Favipiravir complex (PDB code: 4KN6), PA and Baloxavir marboxil complex (PDB code: 6FS6), and PB2 and pimodivir complex (PDB code: 7AS0). The crystal structures were obtained from the Protein Data Bank (PDB) and rendered using PyMol software.

Arbidol (Umifenovir) was first reported in 1993, a small indole derivative [Fig. 2(7) and 3(1)] (51). It was subsequently approved for the prevention and treatment of influenza in Russia and China (52). It is a broad-spectrum antiviral drug with activity against a variety of viruses *in vitro* and *in vivo* studies, including Zika virus, flaviviruses, Ebola, SARS-CoV-2, etc. (53–56). Arbidol primarily targets IAV and IBV, and its epitope is located in the hydrophobic groove of the HA-Stalk, approximately ~16 Å from and interacting with the fusion peptide (57). It blocks the low pH-mediated membrane fusion process by stabilizing the pre-fusion conformation of HA (58).

**Fig 3:**
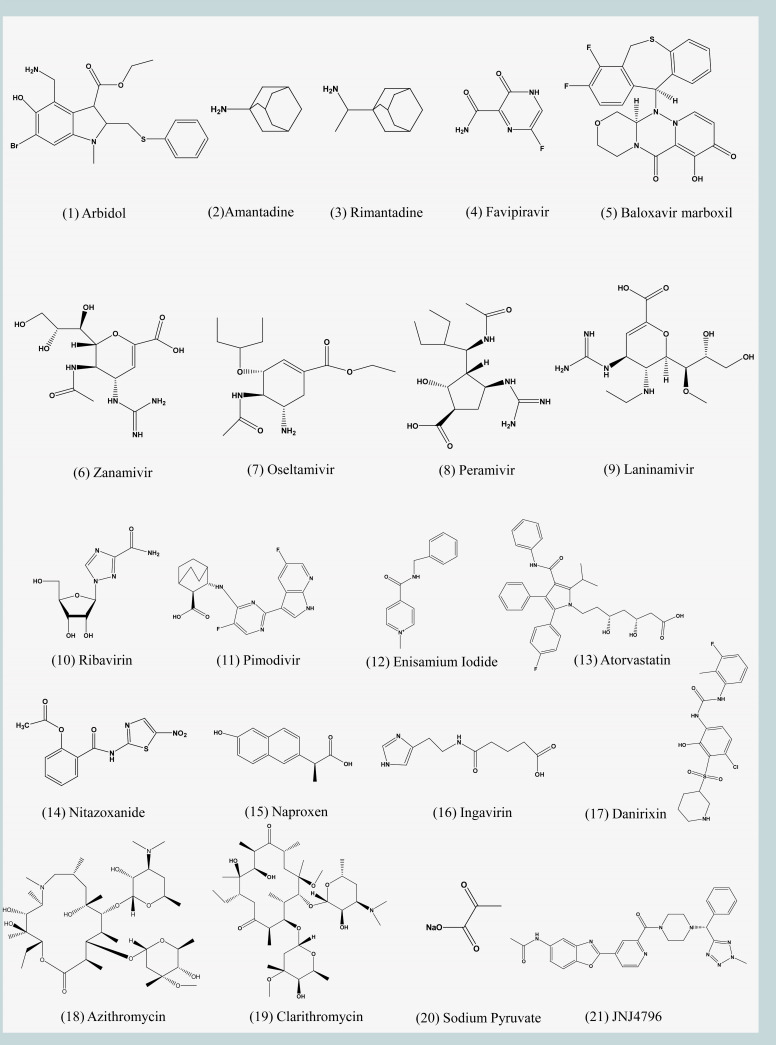
Chemical structures of IV small molecule drugs. The chemical structures of related small molecule drugs were drawn using Chemdraw software.

*In vitro* experiments revealed that the 50% inhibitory concentration (IC_50_) of arbidol on IAV is 4.4–12.1 μM (59). In addition, another study showed that arbidol is equally sensitive and unlikely to develop resistance to 18 clinically isolated strains of IAVs circulating in 2012–2014 seasons (60). In a mouse model, arbidol inhibited weight loss and reduced pulmonary viral load at 25 mg/kg. Also, the treatment of ferrets with arbidol down-regulated several influenza-induced proinflammatory cytokines (IL-10, TNF-α, IL-8, and IL-6) (59). Several clinical studies have shown that arbidol is effective in inhibiting influenza and can lower the patient’s body temperature with minimal side effects (61, 62). Oral administration of arbidol 200 mg four times daily for 5 days to adult influenza patients significantly relieved symptoms after 60 hours, which was able to significantly promote viral shedding (63). Recent data indicated that arbidol is most effective in treating the acute phase of influenza in adults (64).

#### M2 channel inhibitors

Amantadine (trade name generic) and its methyl derivative rimantadine (Flumadine and generic) are the earliest antiviral drugs approved by the U.S. FDA for the prophylaxis and therapy of IAV [Fig. 2(1)(2) and 3A(2) (10]. Amantadine and rimantadine bind to the M2 protein and inhibit ion channel activity, thereby preventing the acidification of the viral core, a key step for viral entry. In the absence of viral core acidification, vRNP is no longer released from the endosome of infected cells, thus blocking the influenza life cycle (11, 12).

In 12 clinical trials, amantadine proved effective in preventing IAV infection in children, but it required its use in up to 17 children over a period of 14–18 weeks. It is also difficult to distinguish adverse effects caused by the drug, such as nausea and dizziness, from the clinical manifestations of influenza (13, 14). Unlike amantadine, the data are insufficient to determine the preventive effects of rimantadine in children, and it should only be used to assist in reducing fever in children (13). In the elderly group, based on a randomized subgroup of 482 cases, rimantadine was less protective than zanamivir, an NA blocker which we discuss in the subsequent section (13, 15). The efficacy of amantadine in adult patients has been widely recognized, but due to pronounced intestinal adverse reactions, it may produce more serious adverse reactions of the central nervous system, and the treatment dose is close to the dose of side effects, therefore, it is difficult to promote in clinical application (16).

At the same time, during prolonged and frequent treatment with amantadine and rimantadine, corresponding drug-resistant viruses are produced, mainly H1N1 and H3N2 subtypes that constitute seasonal influenza, and the proportion of drug-resistant strains increases year by year (17–19). Therefore, these inhibitors are no longer recommended by the US CDC for the treatment or chemoprophylaxis of IAV currently circulating (10).

### Replication inhibitors (RdRp inhibitors)

RdRp is considered to be one of the most promising targets against RNA viruses and is the basis for the current design of the COVID-19 drugs Remdesivir and Molnupiravir (132, 133). RdRp is a key enzyme for the replication and transcription of the genetic material of RNA viruses. RdRp is also highly conserved in different subtypes of IVs (42, 43, 65, 134). Therefore, RdRP inhibitors may be one of the hot spots for future influenza drug development.

Favipiravir (T-705, trade name Avigan) is a purine nucleoside analog that has been shown to inhibit IV transcription and replication by competing with substrates of PB1 (66). The mechanism of action of favipiravir differs from approved influenza antivirals in that antiviral activity against H5N1 and H7N9 avian IVs has been demonstrated in non-clinical data (65, 67). As a result, favipiravir was approved in Japan in 2014 for the treatment of novel or re-emerging IV in view of the outbreak of influenza infection at that time. Subsequently, favipiravir also proved to be a broad-spectrum inhibitor that is against IAV, IBV, and ICV [Fig. 2(8) and 3(4)] (67, 68). Because the structure and function of PB1 are similar in different viruses, it is also used to treat respiratory syncytial virus, Ebola virus, and SARS-CoV-2 infection (69–71). Favipiravir is usually used in combination with oseltamivir in clinical trials of severe influenza (72, 73). The latest research by Bin Cao’s team at China-Japan Friendship Hospital showed that the combination of favipiravir and oseltamivir was more effective in reducing the duration of illness in patients with severe influenza than oseltamivir monotherapy (72). Currently, two clinical trials of favipiravir for the treatment of uncomplicated influenza in adults have been completed (NCT02026349 and NCT02008344). Both trials found that favipiravir significantly reduced viral titers, but the time to symptom relief compared to placebo was different in the two trials, at 14.4 hours and 6.1 hours. While this may be accounted for by a variety of factors including drug dose, greater benefit may be obtained by increasing the dose of favipiravir or by combining it with other drugs (135).

Baloxavir marboxil (S-033188, trade name Xofluza) is developed by Shionogi & Co., Ltd. and Roche for the treatment of IAV and IBV [Fig. 2(9) and 3(5)] (42). It was approved by the FDA in October 2018 for the treatment of acute uncomplicated influenza (influenza) in patients 12 years of age and older who have had symptoms for 48 hours or less (44). In 2022, the population is expanded to include pediatric patients 5–11 years of age who are symptomatic for less than 2 days with influenza (136). As the latest anti-influenza drug, baloxavir marboxil acts differently from its predecessors in that it targets the cap-dependent endonuclease in PA subunit, preventing viral mRNA transcription and thereby inhibiting IV replication (42, 43). PA/I38 is the main binding site for baloxavir marboxil, which can lead to the development of drug resistance when mutations occur (45). Preclinical models showed that compared with oseltamivir and favipiravir, baloxavir marboxil had higher inhibitory activity against IAV and IBV; however, in viruses with PA I38T, the van der Waals force between the drug and the epitope was significantly weakened, and the inhibitory activity was greatly reduced (46). Baloxavir marboxil is safe for the treatment of uncomplicated influenza in adults and adolescents, and it can significantly shorten the duration of influenza symptoms, which is better than oral oseltamivir (47). Notably, baloxavir marboxil and oseltamivir are comparable in high-risk outpatients, but combining the two drugs in routine situations is not recommended (48, 49). Furthermore, pharmacokinetic analysis of three different populations showed that body weight and ethnicity were the most important factors affecting the clearance rate and distribution volume of baloxavir marboxil (50). Currently, studies in China and Korea have shown that baloxavir dosing regimens are appropriate, and the pharmacokinetics and safety profile are consistent in Asian populations (137, 138).

### Neuraminidase inhibitors

To date, three NA inhibitors (zanamivir, oseltamivir, and peramivir) have been approved by the FDA. Laninamivir was approved for influenza treatment in Japan in 2010. While no single study directly compared the efficacy of all four drugs, oseltamivir remains the drug of choice with a safer, broader trial population that includes pregnant women and infants.

Zanamivir (trade name Relenza) is the first NA inhibitor to be approved by the FDA. It is a sialic acid and its transition state analog. Based on 2-deoxy-2,3-didehydro-N-acetylneuraminic acid (DANA, which has been shown previously to inhibit bacterial, viral, and mammalian neuraminidases), it replaces 4-hydroxyl into 4-guanidino group, which increases the binding area of zanamivir to the enzyme activity center [Fig. 2(3) and 3(6) ] (20, 21). It is an inhaled powdered drug mainly used for IAV and IBV; however, the powdered nature of the drug is contraindicated in patients with underlying respiratory disease (22). IV replication peaks between 24 and 72 hours after onset, so drugs that act during the replication phase of the virus, such as NA inhibitors, should be used as early as possible (23). Clinical data show that zanamivir reduced the duration of illness by inhalation in adults and had no significant improvement in children (24, 25). However, zanamivir is safe and effective in the intravenous treatment of children infected with influenza, and there are no significant complications (26). Therefore, zanamivir is primarily suitable for adults and children older than 7 years of age who have contracted acute influenza within 2 days.

The design of oseltamivir (trade name Tamiflu) is also based on DANA, which was approved by the FDA in 1999 as an oral treatment for uncomplicated influenza [Fig. 2(4) and 3(7)] (27–29). It inhibits the activity of NA and prevents the emergence, replication, and infection of the virus, mainly responsible for the treatment and prevention of infection with IAV and IBV (27). Numerous clinical trials have shown that oseltamivir is sufficiently safe for influenza in adults (30, 31). Early treatment with oseltamivir in adult hospitalized patients can shorten fever and hospitalization and reduce the risk of complications but may cause nausea and vomiting (31). Oseltamivir is also recommended for early use in hospitalized children, and in an evaluation of 55,799 hospitalized pediatric patients, it was shown to be effective in reducing clinical symptoms produced by influenza when administered early in the course of illness (139). The CDC reported that antiviral treatment within 48 hours of the onset of influenza provides the greatest clinical benefit (10).

After the 2009 influenza pandemic, oseltamivir was also approved for treatment of infants aged >2 weeks, but there is still insufficient clinical data to demonstrate the safety and efficacy of oseltamivir (32). Kimberlin et al. determined the dose of oseltamivir, in children aged <2 years, twice daily is 3.0 mg/kg for infants aged 8 months and 3.5 mg/kg for infants aged 9–11 months. In clinical trials of oseltamivir in the treatment of influenza in children, it has been shown that oseltamivir can significantly shorten the duration of illness and reduce the incidence of otitis media complications (28, 33). Current seasonal influenza has very low resistance to NA inhibitors compared to amantadine, but the CDC suggests that this may change (10). Studies have shown that oseltamivir A(H1N1) resistant strains generally include the H275Y mutation (34, 35).

Peramivir (BCX-1812, RWJ-270201, trade name Rapivab) is a novel cyclopentane sialic acid analog that has been designed to target NA with inhibitory activity against IAV and IBV [Fig. 2(5) and 3(8)] (20, 36). Unlike oseltamivir, peramivir is less effective when taken orally and is therefore administered intravenously, but it is also the best option for young infants unable to swallow pills (10). Early treatment with peramivir can effectively reduce mortality (37). It was approved by the FDA in 2017 for the treatment of uncomplicated influenza in adults and children from the age of 2 years (38). The clinical efficacy of peramivir has been demonstrated to be safe and effective in several studies (37, 39, 40). Recently, a preliminary randomized controlled trial showed that intravenous peramivir and oral oseltamivir were equally effective in the treatment of emergency uncomplicated influenza patients with similar complications (37, 41). Oseltamivir was significantly more effective than paramivir in clinical studies for the treatment of hospitalized children infected with IAV, while studies in the treatment of infected IBV have shown similar results for both (140).

Laninamivir (R-125489) is an NA inhibitor obtained by replacing the 7-methoxy of zanamivir with 7-hydroxyl [Fig. 2(6) and 3(9)] (74). Laninamivir Octanoate (CS-8958) is a prodrug of laninamivir, which is inhaled and converted into laninamivir through the respiratory tract (75, 76). Both drugs were approved in Japan in 2010 and are currently undergoing clinical trials in other countries (75, 77, 78). Laninamivir is a long-acting inhibitor with a half-life of up to 3 days and no significant adverse events (79). Based on recently updated data, a single inhalation of 160 mg has been recommended for the treatment of patients over 5 years of age in Japan (80). Double-blind, multicenter, randomized, placebo-controlled studies show that a single inhaled dose of 20 mg laninamivir octanoate is effective and well-tolerated in the treatment of influenza in adults and children (78, 81). Other studies have reported that laninamivir octanoate is comparable to oseltamivir and zanamivir in the treatment of influenza and significantly reduces the duration of treatment in children infected with oseltamivir-resistant A(H1N1) viruses carrying H274Y NA substitution (82, 83).

## DRUGS IN THE CLINICAL TRIALS

### Polypeptide or small molecule drugs

#### Entry inhibitors

DAS181 (Fludase) is an inhaled recombinant protein, which is composed of Actinomyces viscosus sialidase catalytic domain and a cell surface anchored sequence. It accurately removes sialic acid from the epithelial cells of the respiratory tract, a receptor for parainfluenza, IV and other respiratory viruses (Fig. 1; Table 3) (88). In this way, multiple subtypes of seasonal and avian influenza were effectively inhibited with 50% effective concentration (EC_50_) values ranging from 0.04 nM to 0.9 nM. DAS181 showed positive preventive and protective effects in mice and ferrets (88). In addition to acting on the upper respiratory tract, it also prevents the H5N1 IV from infecting human lung tissue. Data from an *in vitro* study showed that DAS181, administered at 100 U/mL every 12 hours, produced the most effective inhibitory activity (88).

A double-blind, placebo-controlled phase II clinical trial (NCT01037205) evaluated the efficacy and safety of DAS181 in patients primarily infected with seasonal influenza. Compared to the placebo group, viral load was significantly lower in both the multi-dose group (10 mg daily for 3 days) and the single-dose group (10 mg) pharyngeal washout, but testing at 3 or 5 days later found that only the multi-dose group significantly reduced patients’ viral load (89). The study showed that the major side effect of DAS181 was the elevation of alkaline phosphatase in the blood (89). Other studies (NCT00527865 and NCT01651494) found that DAS181 was not suitable for continuous treatment beyond 7 days because of adverse effects, such as dyspnea, inducing specific neutralizing IgG antibodies and rendering the drug ineffective (90).

#### Replication inhibitors

Ribavirin (Virazole), a synthetic purine nucleoside analog, is a broad-spectrum viral inhibitor approved for the treatment of hepatitis C and respiratory syncytial virus [Fig. 3(10)] (141, 142). It also has inhibitory activity against IV, acting on the PB1 subunit of RNA polymerase and blocking the synthesis of viral mRNA (8). Ribavirin and several drugs have shown synergistic effects against IV both *in vivo* and *in vitro*, including baicalein, favipiravir, oseltamivir, and amantadine (103–106). Preclinical data showed that the combination of ribavirin, oseltamivir, and amantadine exhibited high inhibitory activity against multiple IV, including drug-resistant strains, and was superior to oseltamivir alone. The pharmacokinetics (NCT00867139) of these three drugs (200 mg, 50 mg, and 75 mg, respectively) are similar to monotherapy and are safe for use in immunocompromised patients (107). Results from a randomized, double-blind, multicenter phase II clinical trial (NCT01227967) showed that despite a significant reduction in viral output at 72 hours compared with monotherapy, this difference was not associated with improved clinical benefits (108). Therefore, more data are needed for analysis and discussion to identify the effectiveness of Ribavirin.

Pimodivir (JNJ-63623872, VX-787) is an indole analog that interacts with the cap-binding domain of the PB2 subunit to block viral mRNA transcription [Fig. 2(10) and 3(11)] (109). Pimodivir has excellent inhibitory activity against IAV, including H1N1 pdm09, H3N2, and H5N1 subtypes, as well as viruses resistant to oseltamivir and amantadine., with EC_50_ values of 0.13–3.2 nM (110). *In vivo*, 20 mg/kg twice daily doses of pimodivir completely prevented H3N2 and H1N1 infection in mice. Therefore, it shows more effectiveness than the same dose of oseltamivir (111). The elderly are always the most vulnerable group to influenza. In a phase II clinical study (NCT02532283) evaluating pimodivir combination treatment with oseltamivir IAV pharmacokinetic in different groups, the result showed that pimodivir pharmacokinetic parameters were similar in non-elderly and elderly patients, and combined treatment can reduce complications and sick time, better than that of oseltamivir monotherapy (112). In a double-blind IIb study (NCT02342249) evaluating the efficacy of pimodivir in the treatment of uncomplicated acute influenza in adults, viral load was lower with pimodivir (300 mg and 600 mg) or the combination of pimodivir and oseltamivir (600 mg + 75 mg) compared to the placebo group (113). However, the Phase III data (NCT03376321) did not demonstrate an additional benefit of pimodivir monotherapy in hospitalized patients with IAV, and the clinical development trial was subsequently terminated.

Enisamium iodide is an anti-respiratory viral (including IAV and IBV) drug that acts on viral RNA polymerase via its metabolite VR17-04 [Fig. 3(12)] (114). In the primary normal human bronchial epithelial cells, 500 µg/mL of the drug significantly inhibited H1N1 replication, and 200 mg/kg enisamium iodide daily could cure ferrets infected with 10^5^ TCID_50_ of H3N2 and reduced respiratory viral load (115). A phase II trial evaluated the efficacy of enisamium iodide in patients aged 18–60 years with confirmed influenza and showed a reduction the viral load and reduced duration of illness on day 3 of treatment compared to placebo (114). Currently, enisamium iodide is used to treat influenza in countries such as the Commonwealth of Independent States and Mongolia (115).

Atorvastatin is a 3-hydroxy-3-methylglutaryl-coenzyme A reductase inhibitor that effectively reduces plasma cholesterol levels [Fig. 3(13)] (91). The envelope of IV contains a large amount of cholesterol, and Atorvastatin can effectively inhibit the synthesis of cholesterol in lipid droplets caused by infection stress in cells, thereby affecting IV replication (92). A phase II trial (NCT02056340) is currently being completed in Israel to evaluate Atorvastatin in patients hospitalized for acute influenza. However, follow-up results have not yet been published.

#### Assembly inhibitors

Nitazoxanide is the only FDA-approved drug for the treatment of Cryptosporidium infection and also has inhibitory effects against intestinal parasites such as helminths and tapeworms [Fig. 3(14)] (93). Later, Nitazoxanide was found to have broad-spectrum antiviral activity against influenza, SARS-CoV-2, MERS-CoV, paramyxovirus, and norovirus (94–97). The IV progeny viruses require complete assembly of the progeny virions at the cell membrane surface, but nitazoxanide blocks HA terminal glycosylation and affects HA transport between the endoplasmic reticulum and the Golgi complex, thereby inhibiting the maturation of HA glycoproteins and the assembly of the IV (98). Significant inhibitory activity of nitazoxanide against H1N1, H3N2, and IBVs prevalent between March 2014 and August 2016 was demonstrated using plaque reduction assays (99). Preclinical studies have shown that nitazoxanide in combination with oseltamivir is more effective than monotherapy in the prevention of H1N1 infection, but substantial clinical data are needed for nitazoxanide in combination with other drugs (100). In a Phase IIb/ III trial (NCT01227421), 600 mg twice daily nitazoxanide treatment for 5 days reduced symptom duration in patients with acute uncomplicated influenza, and headache was the main and common adverse event reported in different groups (101). However, a recent clinical trial (NCT02057757) in patients with severe acute influenza reflected that the effect was not satisfactory (102).

Naproxen is a non-steroidal anti-inflammatory drug agent that inhibits IAV and IBV *in vitro* and *in vivo*. Related studies have shown that 10 mg/kg daily of Naproxen is more effective than the equivalent dose of oseltamivir in treating mice infected with IBV [Fig. 3(15)] (116). The host export protein CRM1 binds to the NP and mediates the export of the RNP complex from the nucleus. The mechanism of action suggests that Naproxen recognizes NP and inhibits the binding of CRM1 to NP protein through antagonism, thus inhibiting the NP output of IV. The critical residues on IAV NP and IBV NP were different, Y148 and F209, respectively (116). A recent clinical trial (NCT04315194) conducted in adult hospitalized patients with H3N2 infection revealed that combined therapy of naproxen (200 mg)-oseltamivir (75 mg)-clarithromycin (500 mg), compared with the control arm of oseltamivir (75 mg) monotherapy, reduced 30-day mortality by 7.3%, supporting the use of multi-drug-combined therapy (117).

#### Other

The following drugs are currently in the late clinical stage or have been approved for the treatment of influenza in Russia. Despite the lack of relevant clinical data and literature, they are still shown for reference only. See www.clinicaltrials.gov for details. XC221 is an innovative drug, and a phase II clinical trial (NCT03455491) has been completed in Russia to evaluate the efficacy and safety of XC221 in the treatment of IV or other acute respiratory virus infections (SARS-CoV-2). Ingavirin (Imidazolyl ethanamide pentandioic acid), a drug developed by Valenta, a Russian pharmaceutical company, inhibits IV by blocking the transport of newly synthesized NP from the cytoplasm to the nucleus [Fig. 3(16)] (84). Literature has shown that Ingavirin can effectively inhibit influenza subtypes such as H1N1, H3N2, H5N1, and B *in vivo* or *in vitro* (85–87). Currently, a phase IV clinical trial (NCT03154515) has been completed.

### Monoclonal antibody drugs

Monoclonal antibody drugs against IV have higher affinity and inhibitory activity than small molecule drugs (143). The current clinical antibody epitopes are different from the small molecule drugs in the market, which has application value in the development of broad-spectrum influenza vaccine and drug combination (144). Currently, monoclonal antibody drugs involved in clinical studies are mostly derived from vaccinated sera of healthy volunteers or recovered patients, except TCN-032, all of which are directed against the stalk domain of the HA. Importantly, these antibodies can inhibit multiple IV subtypes *in vitro* and *in vivo* (145). Due to its functional specificity, the stalk domain has a significantly lower mutation rate than the Head, which leads to the antibodies or compounds targeting the Stalk are usually highly broad-spectrum (146). Therefore, the antibodies targeting the Stalk are expected to become the basis for the research on universal vaccines (147, 148). However, subsequent clinical results have shown that the efficacy of these antibodies as monotherapy is generally unsatisfactory, and the direction of adjuvant combination therapy should be considered in the future (Fig. 1; Table 3).

**Fig 4:**
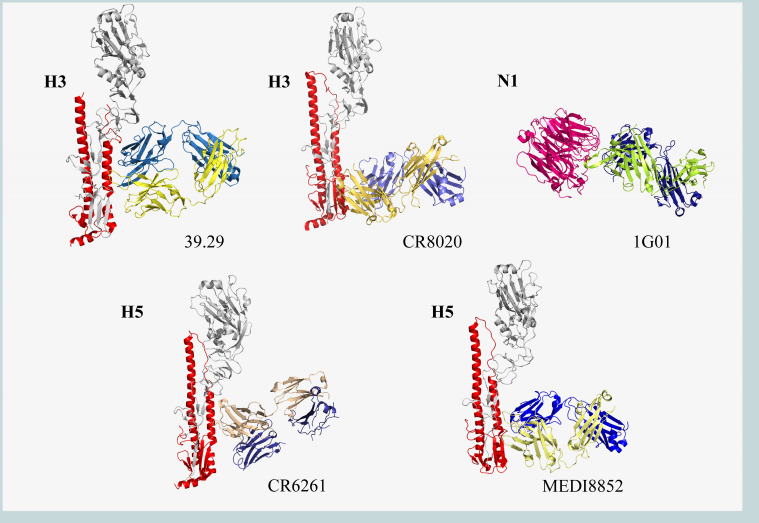
Crystal structures of antibodies against IV glycoprotein. The HA monomers (gray and red) bind to the heavy (blue) and light (yellow) chains of the antibodies. MHAA4549A (39.29; PDB code: 4KVN), CR8020 (PDB code: 3SDY), CR6261 (PDB code: 3GBM), MEDI8852 (PDB code: 5JW4), and 1G01 (PDB code: 6Q23). Graphics acquisition and rendering methods are the same as Fig. 2.

CR6261 is a human broad-spectrum neutralizing antibody screened by phage display technology (Fig. 4; Table 4). It neutralizes H1, H2, H5, H6, H8, H9, H13, and H16 subtypes *in vitro* with IC_50_ values of 0.12–14.87 μg/mL (149, 150). Meanwhile, CR6261 showed a cross-protective effect in both prevention and treatment of H5N1 and H1N1 infection in mice (149). The heavy chain variable region of CR6261 is encoded by the IGHV1-69 gene, which can recognize the conserved hydrophobic pocket on HA-Stalk and prevents low pH-mediated HA conformational changes, thus inhibiting the fusion of viral and host membranes (151). Although preclinical trials have shown promising prophylactic and therapeutic efficacy, in a subsequent randomized, double-blind, placebo-controlled phase II trial (NCT02371668) in which 50 mg/kg of CR6261 was administered to healthy volunteers at 24 hours after H1N1 infection, no significant prophylactic or therapeutic effects were reported for monotherapy CR6261 (152, 153).

**TABLE 4:** Influenza drugs in clinical trials (monoclonal antibody drugs)

Name	Binding to	IC_50_ (μg/mL)	100% protection *in vivo*	Adverse event	Usage	Clinical studies	Reference
CR6261	HA-stalk (H1, H2, H5, H6, H9, H13, and H16)	0.12–14.87	Pre: 5 mg/kg Post: 15 mg/kg	−^*a*^	Intravenous	I NCT01406418, February 2013 to November 2013; II NCT02371668, February 2015 to November 2018	(149–153)
CR8020	HA-stalk (H3, H4, H7, H10, H14, and H15)	1.1–13.1	Pre: 3 mg/kg Post: 15 mg/kg	−	Intravenous	I NCT01756950, January 2013 to November 2013; II NCT01938352, October 2013 to January 2014	(150)
VIS410	HA-stalk (H1, H2, H3, H5, H6, H7, and H9)	0.3–11	Post: 2.5 mg/kg	Mild diarrhea	Intravenous	I NCT02045472, September 2014 to May 2015; II NCT02989194, January 2017 to October 2017	(154–157)
MHAA4549A	HA-stalk (H1, H2, H3, H5, and H7)	1.3–45.1	Post: 100 and 900 µg	Headache	Intravenous	I NCT01877785, July 2013 to November 2013; I NCT02284607, November 2014 to March 2015; IIa NCT01980966, November 2013 to June 2014; IIb NCT02293863, January 2015 to May 2017	(158–161)
MEDI8852	All HA-stalk	0.064	Pre: 1 mg/kg Post: 10 mg/kg	Headache, hypoglycemia, and bronchitis	Monotherapy	I II NCT02350751, December 2015 to December 2016	(162, 163)
TCN-032	M2e (H1, H2, H3, H5, H7, andH9)	−	−	−	Monotherapy	I NCT01390025, September 2011 to March 2012; II NCT01719874, August 2012 to March 2013	(145, 164)
1G01	N1-N9 NA and IBV NA	0.01–2	Pre: 0.3 mg/kg Post: 5 mg/kg	−	−	−	(129)

^*a*^ "−" indicates that no relevant information is available at this time of publication.

The same approach was used to screen the H3-HA responding monoclonal antibody CR8020, which has now completed a phase II clinical trial (NCT01938352) evaluating intravenous therapy, but no results have been published (Fig. 4; Table 4). The initial studies showed that CR8020 neutralized the H3, H7, and H10 subtypes *in vitro* with IC_50_ values of 1.1–13.1 μg/mL and complementation with CR6261 to neutralize almost all subtypes of IAV. *In vivo*, 3 mg/kg of CR8020 could effectively prevent and protect mice from infection with lethal doses of H3N2 and H7N7 (150). Both CR8020 and CR6261 target HA-Stalk; the difference is that CR8020 acts near the proximal end of the viral membrane and inhibits the conformational changes of HA and the membrane fusion process by stabilizing the structure of the fusion peptide (150). VIS410 targets HA-Stalk and broadly neutralizes the H1, H3, and H5 subtypes of IAV *in vitro* with EC_50_ of 0.3–11 μg/mL (Table 4). A single therapeutic dose of VIS410 protected BALB/c and DBA mice infected with H3N2 and H7N9, respectively (154). Moreover, a synergistic effect was demonstrated in combination with oseltamivir. VIS410 inhibited the viral membrane fusion process, targeted the elimination of infected cells through antigen-dependent cell-mediated cytotoxicity, and controlled IV-induced acute respiratory distress symptoms (154, 155). Given the good preclinical trial data support, VIS410 was quickly followed up with relevant studies. Phase I clinical trial (NCT02045472) has demonstrated that VIS410 at 50 mg/kg is safe, well tolerated, with no significant adverse reactions, and has good therapeutic benefits for the hospitalized population over 65 years of age (156). In a Phase II trial (NCT02989194), a single intravenous dose of 2,000 mg or 4,000 mg was effective in reducing viral load in the nasopharynx of patients with uncomplicated influenza compared with placebo (157). The main adverse reaction of treatment was mild diarrhea, which was dose-dependent (157). This provides strong support for the development of monoclonal antibodies to treat and prevent severe influenza infections.

MHAA4549A (39.29) can neutralize the H1 and H3 subtypes with IC_50_ of 1.3–45.1 nM (Figure, Table 4). MHAA4549A was more effective than oseltamivir in mice and ferrets infected with H1N1 and H3N2 (158). Furthermore, a synergistic effect was noted when used in combination with oseltamivir. The crystal structure shows that MHAA4549A binds to the top of highly conserved stalk helix A (158). Subsequently, two phase I randomized, double-blind, and placebo-controlled clinical trials (NCT01877785 and NCT02284607) confirmed the safety and tolerability of MHAA4549A in healthy volunteers, with no serious adverse events and no occurrence of immunogenicity (159). Although MHAA4549A also proved to be well tolerated and significantly reduced influenza-related symptoms in phase II clinical trial (NCT01980966), MHAA4549A did not significantly shorten the time to normal respiratory function and was less clinically effective than oseltamivir alone in an interim trial combining MHAA4549A and oseltamivir in patients hospitalized with severe influenza (NCT02293863), and the trial was subsequently discontinued (160, 161).

MEDI8852 is a broad-spectrum human mAb effective against H1, H2, H3, H5, H6, H7, and H9 subtypes with mean EC_50_ of 0.064 µg/mL (Fig. 4; Table 4) (162). It has higher antibody neutralization breadth and potency than other monoclonal antibodies, and in mouse and ferret models, MEDI8852 acts in a dose-dependent manner, showing a strong therapeutic effect that was superior to oseltamivir (162). MEDI8852 binds to the hydrophobic groove and most of the fusion peptides by coordinating the movement of its own heavy- and light-chain complementary variable regions (162). However, in the Phase IIa trial (NCT02603952), MEDI8852 produced slightly more adverse events than the oseltamivir group and did not demonstrate clinical significance due to the small number of participants in the studies of treatment alone and in combination with oseltamivir. Therefore, the company decided to discontinue the follow-up trial (163).

Most of the influenza antibody drugs currently in clinical use target HA, while TCN-032 is the only monoclonal antibody that targets the M2 extracellular region (highly conserved in IAV) (Fig. 4; Table 4) (145). Although M2-targeting antibodies have no neutralizing effect, they inhibit the emergence and assembly of the virus by binding to M2 (165). The antibody was obtained from B cells of healthy volunteers, and *in vivo* experiments showed that TCN-032 ensured the survival of mice infected with lethal doses of H5N1 or H1N1 and helped them regain weight rapidly (145). TCN-032 has been shown to be well tolerated and pharmacokinetic (NCT01719874) in healthy people without immunogenicity or other significant side effects, but in a phase II intravenous evaluation (NCT01719874), TCN-032 was not able to significantly reduce influenza-related symptoms (164).

## POTENTIAL FUTURE DIRECTION

Combination therapy is commonly used in clinical trials, where a combination of two or three drugs with different mechanisms of action at a safe and effective dose can provide a more comprehensive inhibition. For example, the combination of antibodies CR6261 and CR8020 neutralizes all subtypes of IAV and oseltamivir in combination with anti-inflammatory drugs reduces the duration of influenza symptoms. Paxlovid, a COVID-19-specific drug consisting of Nirmatrelvir-Ritonavir, uses Ritonavir (a protease inhibitor and cytochrome P450 3A inhibitor) to inhibit key drug metabolism enzyme cytochrome P450 and increase the plasma concentration of Nirmatrelvir (a main protease inhibitor), preventing viral replication (166). In addition, frequent single dosing may lead to the development of drug-resistant strains of influenza, whereas combination therapy with influenza drugs with different mechanisms of action would greatly reduce the selection pressure of drug-resistant viruses (167, 168).

Treatment of uncomplicated influenza can be treated with drugs that target the virus itself, while serious acute IV infection can lead to pneumonia and acute respiratory failure, causing concurrent bacterial infection (169). Therefore, relevant anti-inflammatory drugs are needed to reduce the occurrence of influenza-related complications in order to shorten the time of illness of influenza patients. Severe acute influenza has been shown to trigger an overreaction of neutrophils, resulting in alveolar damage and increased viral load (170). Danirixin (GSK1325756) is a reversible and selective CXC chemokine receptor 2 (CXCR2, the key receptor for neutrophils to chemotaxis to inflammatory sites) antagonist that blocks neutrophils’ chemotaxis in inflammation, thereby reducing excessive inflammatory response [Fig. 3(17)] (171). Macrolides are a class of antimicrobial antibiotics that act on bacterial ribosomes and inhibit protein synthesis. They also regulate the body’s immunity function and eliminate excessive inflammatory response [Fig. 3(18)(19)] (172). Sodium pyruvate (the exogenous form of pyruvate) has been shown to have powerful anti-inflammatory and antioxidant effects [Fig. 3(20)]. It can reduce the immune response by inhibiting the production of reactive oxygen species in mitochondria and effectively reduce the related inflammatory cytokines caused by IV (173). These drugs have been clinically involved in the adjuvant treatment of patients with influenza infection.

The emergence of COVID-19 has once again demonstrated that it is difficult to develop specific drugs against outbreak viruses in a timely manner, and only extensive screening of existing drugs can shorten the time to clinical use. Due to the particularity of the mechanism of action, multiple drugs possess cross-inhibitory activity against viruses, making them candidates for the inhibition of multiple viruses. Ribavirin, an RNA polymerase inhibitor approved for the treatment of hepatitis C and respiratory syncytial virus, has also been reported to have inhibitory activity against the IV, SARS-CoV-2 (174). Baloxavir marboxil, the newly approved influenza drug, and pimodivir, developed by Johnson & Johnson, are also RNA polymerase inhibitors with potential cross-inhibiting activity. In addition, Molnupiravir, a novel coronavirus drug developed by Merck Sharp & Dohme, also acts on RNA polymerase and leads to the accumulation of errors in the genome of SARS-CoV-2 through its metabolites, thereby inhibiting replication. This mechanism of action may be applicable to various viral polymerases (175). Thus, the RNA polymerase of IV may become a major choice for future-targeted drugs.

Influenza drugs targeting other sites are also under development, including full-human antibody 1G01 targeting NA [Fig. 3(19)(20) and 4], and oral small-molecule drug JNJ4796 [Fig. 2(11) and 3(21)], which was screened with CR6261 target as a reference, showed strong and extensive binding inhibitory activity *in vitro* and *in vivo* experiments (118, 129). These drugs are ideal clinical candidates. Ignoring the virus will not cause it to disappear. Under the premise of the decline of the threat to human health by SARS-CoV-2, IV has returned to the public view, causing various concerns, including the current widespread seasonal influenza, frequent outbreaks in poultry and occasional cross-species transmission of H5 subtype avian influenza outbreaks. We all need to respond promptly and effectively.

## CONCLUDING REMARKS

IV has coexisted with humans for millennia. While significant efforts have been made on influenza antiviral drug development, influenza infection still poses a severe public health burden due to drug resistance problems, such as with amantadine and rimantadine. As mentioned above, selecting combinations of drugs with different mechanisms of action in response to drug failure due to viral mutations may be the main way to combat IVs in the future. Another way to mitigate drug resistance issue is by designing drugs that target core structural components or enzymatic active sites of viral proteins, as mutations in these residues likely produce structurally unstable or inactive protein. In addition, there are many druggable nodes in the IV life cycle that are necessary for virus-host interactions. To ensure the structural stability and function of related proteins, even if resistance mutations occur, there are still a large number of other non-mutated sites as alternatives that would be good drug targets and would minimize the problem of drug resistance. It is worth mentioning that drugs that have developed serious resistance problems may be used in future emerging infectious diseases.

In summary, the four influenza pandemics are constant reminders that negligence will produce incalculable damage that will continue to affect a generation or even generations. Constant discovery of novel influenza antiviral drug for the prevention of influenza pandemics is indeed essential.

## EDITOR'S NOTE

The American Society for Microbiology and *mBio* would like to inform the readers that this article is a republished version of https://doi.org/10.1128/mbio.01273-23, which was retracted (https://doi.org/10.1128/mbio.00106-24). As stated in the retraction notice, after publication, "A number of factual errors were found in the paper" by the authors. Additionally, "there were some spelling errors and language presentation in the article which may lead to misinterpretation of the content by readers." This led to a number of inaccuracies in the minireview, too many to be addressed through a correction. Therefore, the authors were allowed to submit an entire, corrected manuscript for consideration to the journal. The manuscript was editorially reviewed and subsequently accepted.
